# The Elevation and Impact of Peripheral Bile Acids in Chronic Lymphocytic Leukemia

**DOI:** 10.3390/biomedicines13040874

**Published:** 2025-04-04

**Authors:** Audrey L. Smith, Abigail Ridout, Sydney A. Skupa, Rolando Martinez-Rico, Erin M. Drengler, Eslam Mohamed, Christopher R. D’Angelo, Dalia El-Gamal

**Affiliations:** 1 Eppley Institute for Research in Cancer and Allied Diseases, University of Nebraska Medical Center, Omaha, NE 68198, USA; aridout1911@csm.edu (A.R.); sydney.skupa@unmc.edu (S.A.S.); rmartinez-rico@unomaha.edu (R.M.-R.); edrengler@unmc.edu (E.M.D.); 2 College of Medicine and College of Graduate Studies, California Northstate University, Elk Grove, CA 95757, USA; eslam.mohamed@cnsu.edu; 3 Division of Hematology and Oncology, Department of Internal Medicine, University of Nebraska Medical Center, Omaha, NE 68198, USA; christopher.dangelo@unmc.edu; 4 Fred and Pamela Buffett Cancer Center, University of Nebraska Medical Center, Omaha, NE 68198, USA; 5 Division of Hematology/Oncology, Department of Internal Medicine, College of Medicine, University of Cincinnati, Cincinnati, OH 45267, USA

**Keywords:** chronic lymphocytic leukemia (CLL), TCL1 mouse model, metabolomics, bile acids

## Abstract

**Background**: Chronic lymphocytic leukemia (CLL) is the most prevalent adult leukemia in the Western world. Targeted therapies have made CLL manageable for many patients, but the ongoing threat of disease relapse or transformation beckons a deeper understanding of CLL pathogenesis, and thus, its durable eradication. This study identifies bile acids (BAs) as elevated in the peripheral blood of CLL patients and a murine model of CLL, in comparison to healthy controls. Elevated BA concentrations have been associated with intestinal malignancies and immunomodulation; however, their role in CLL is relatively unknown. **Methods**: Metabolomic analysis was performed on murine and human plasma. Flow cytometry analysis of CLL patient B-cells and healthy donor T-cells were utilized to evaluate the immunomodulatory impact of differentially abundant BAs. **Results**: Herein, BAs were found to be differentially abundant in CLL. Elevated BAs demonstrated minimal impact on CLL cell proliferation or CLL-associated T-cell function. **Conclusions**: Future studies are needed to determine the mechanistic influence of BAs on CLL pathogenesis.

## 1. Introduction

Chronic lymphocytic leukemia (CLL) is characterized by the abnormal expansion of mature B-cells in the blood and secondary lymphoid organs [1]. As the most prevalent adult leukemia in the Western world, CLL is expected to account for 20,700 new cases and 4440 deaths in the United States in 2025, according to recent SEER projections [2]. CLL incidence has remained stable over the last two decades, but mortality rates have continuously declined, with current 5-year relative survival approaching 88.5%. Although the success of targeted therapy has improved survival outcomes for many patients, CLL is commonly exacerbated by profound tumor microenvironment (TME) immunosuppression, preventing the effective clearance of tumor cells [3]. Metabolic adaptations within CLL cells and the TME play crucial roles in disease progression, with alterations in energy production pathways not only sustaining leukemic cell survival and proliferation but also contributing to immune dysfunction and treatment resistance [4].

Bile acids (BAs) are amphiphilic steroid molecules involved in numerous physiological processes, including digestion and absorption of dietary lipids [5]. Primary BAs [cholic acid (CA) and chenodeoxycholic acid (CDCA)] are generated from the breakdown of cholesterol in the liver and are commonly conjugated with taurine (T) or glycine (G). Primary BAs translocate to the small intestine, where they are deconjugated then dehydroxylated by microbiota bile salt hydrolases to generate secondary BAs [deoxycholic acid (DCA) and lithocholic acid (LCA)] [6]. BAs can pass through the bloodstream via enterohepatic circulation to be recycled through the system [7]. In addition to their role in nutrient homeostasis, BAs are active signaling molecules [8], recognized by receptors such as FXR (primary BAs) and GPBAR1 (secondary BAs). Due to their detergent quality, high BA concentrations (>100 µM) within the gastro-intestinal tract lumen can promote epithelial damage and inflammation-driven carcinogenesis/metaplasia [9,10,11,12]. However, physiological levels (approximately 0.2–5 µM) aid epithelial barrier and immune homeostasis [5]. Interestingly, high levels of BAs are not only locally associated with intestinal and liver pathologies but have also been detected in metastatic tumor-draining lymph nodes [13]. The tumor-supporting properties of BAs may be the result of cellular damage, the activation of receptor signaling, and/or immune-regulatory activity. Several reports have demonstrated the capacity of BAs to alter the function of helper T-cells (e.g., T-regs, Th17 cells) and cytotoxic T-cells [14,15,16]. However, these studies have primarily focused on intestinal immune populations. While little is known about the role of BAs in CLL pathogenesis, their impact on cholestasis, inflammation, and cancer is increasingly being explored.

Herein, we investigated differential metabolites associated with CLL disease, validated elevated BAs in the plasma of CLL patients, and sought to explore how these elevated BAs may influence CLL disease.

## 2. Materials and Methods

A flow chart of the methods used for this study is included as Supplementary Figure S1.

### 2.1. Murine Plasma Metabolomics

Wild type C57BL/6 (WT B6) mice were engrafted with 1 × 10^7^ spleen-derived lymphocytes from a donor Eμ-TCL1 transgenic mouse with advanced disease (adoptive transfer; AT). At 28 days post-engraftment, Eμ-TCL1 AT mice and age-matched WT B6 controls were sacrificed for tissue collection. Blood was obtained via cardiac puncture, then centrifuged at 2000× *g* for 15 min at 4 °C to isolate plasma, which was sent for metabolomic analysis by Metabolon, Inc. (Durham, NC, USA). Samples were prepared using the automated MicroLab STAR^®^ system (Hamilton Company; Reno, NV, USA). Proteins were precipitated with methanol, then subjected to Ultrahigh Performance Liquid Chromatography–Tandem Mass Spectroscopy (UPLC–MS/MS) using acidic positive ion conditions (C18 column, two methods for hydrophilic and hydrophobic compounds) and basic negative ion conditions (two methods: C18 column and HILIC column), as previously described [17]. Data were processed using the Metabolon LIMS system. Following normalization, data were log transformed, and Welch’s two-sample *t*-test was used to identify biochemicals that differed significantly (*p* ≤ 0.05) between Eμ-TCL1 AT and WT samples. Log2 fold change and *p* value data were subject to Ingenuity Pathway Analysis (IPA)-Metabolomics™ (Qiagen; Hilden, Germany; accessed September 2024), and results were obtained as significantly modulated canonical pathways.

### 2.2. Human Plasma Metabolomics

Human plasma samples from treatment-naïve patients with CLL and age-matched healthy donors were sent to Novogene Co. (Beijing, China) for metabolomic analysis. Proteins were precipitated with methanol, then subjected to UPLC–MS/MS with the following specifications—chromatographic column: Waters ACQUITY Premier HSST3 Column 1.8 µm, 2.1 mm × 100 mm; mobile phase A: ultrapure water (0.1% formic acid); mobile phase B: acetonitrile (0.1% formic acid); column temperature: 40 °C; flow rate: 0.40 mL/min; injection volume: 2 µL. Raw data were extracted, peak-identified, quantified, and QC-processed using Novogene’s hardware and software. Welch’s two-sample *t*-test was used to identify biochemicals that differed significantly (*p* ≤ 0.05) between total CLL samples and healthy controls. Log2 fold change and *p* value data were subject to IPA-Metabolomics™ (Qiagen; accessed September 2024), and results were obtained as significantly modulated canonical pathways. One-way ANOVA with Dunnett’s correction for multiple comparisons was used to determine differential BAs between stratified CLL groups and healthy controls.

### 2.3. Colorimetric Detection of Total Bile Acids

Total BAs were detected in undiluted human plasma samples using the Colorimetric Bile Acid Assay Kit from Abcam (Cambridge, UK; Cat #ab239702), and measured on a Tecan Infinite^®^ M1000 Pro microplate reader (Männedorf, Switzerland), following manufacturer protocol.

### 2.4. Reagents

BA salts (Supplementary Table S1) were purchased from Avanti Research (Alabaster, AL, USA), Fisher Scientific (Waltham, MA, USA), Sigma-Aldrich (St. Louis, MO, USA), or MedChemExpress (Monmouth Junction, NJ, USA) and reconstituted in DMSO. The BET inhibitor, JQ1 (Cayman Chemicals; Ann Arbor, MI, USA) was reconstituted in DMSO and used as a control compound known to inhibit malignant B-cell viability and downregulate the expression of immune inhibitory factors [18,19].

### 2.5. Primary Human Cells

T-cells were purified from healthy donor peripheral blood lymphocytes (PBLs; obtained from UNMC Elutriation Core) using the EasySep™ Human T-cell Isolation Kit (StemCell Technologies; Vancouver, BC, Canada). Peripheral blood mononuclear cells (PBMCs) were isolated from CLL patient blood using Lymphoprep™ density gradient medium (StemCell Technologies) according to manufacturer protocols. Patient-derived PBMCs were confirmed to contain >90% CD19^+^/CD5^+^ B-cells prior to experimental use. Healthy donor and CLL patient sample characteristics are listed in Supplementary Tables S2 and S3, respectively.

### 2.6. MTS Cytotoxicity Assay

Primary CLL patient-derived PBMCs (containing >90% CD19^+^/CD5^+^ cells) were plated in 96-well plates at a density of 6–8 × 10^5^ cells/well in complete RPMI-1640 medium, which contains 2 mM L-glutamine (Sigma-Aldrich), 100 U/mL penicillin, 100 μg/mL streptomycin (P/S; Sigma-Aldrich), and 10% heat-inactivated fetal bovine serum (hi-FBS; Avantor^®^; Radnor, PA, USA). Cells were then treated with vehicle (DMSO) or increasing concentrations of BAs for 48 h, unstimulated or in the presence of 1.7 μM CpG-ODN (Integrated DNA Technologies; Coralville, IA, USA). Following treatment, cell proliferation was assessed by MTS assay using the CellTiter 96^®^ AQueous assay kit (Promega, Madison, WI, USA) as specified by the manufacturer, and detailed in a previous report [20].

### 2.7. Co-Cultures

A mixed lymphocyte system [21] was used with minor modifications to optimize induction of T-cell exhaustion. Healthy donor T-cells (1.25 × 10^6^/mL) or healthy donor T-cells co-cultured with CLL B-cells at a 2:1 (B:T) ratio were plated in complete RPMI-1640, stimulated with 1.7 μM CpG-ODN, 10 μg/mL plate-bound anti-CD3 (clone UCHT1, BioLegend; San Diego, CA, USA), and 5 μg/mL anti-CD28 (clone CD28.2, BioLegend) as appropriate, and treated with the indicated BAs or vehicle (DMSO) for 48–96 h. To detect T-cell proliferation, T-cells were labeled with 2 μM Cell Trace™ CFSE (Invitrogen; Waltham, MA, USA) prior to culture. To detect intra-cellular cytokines, following 48 h culture, T-cells were stimulated with 1× PMA/ionomycin for 6 h, with 1× Brefeldin-A (BioLegend) added for the final 5 h. Cells were then evaluated via flow cytometry.

### 2.8. Flow Cytometry

Cells (~1 × 10^6^) were incubated in 100 μL PBS 2% hi-FBS containing fluorochrome-labeled antibodies for 30 min at 4 °C. For the detection of intra-cellular and intra-nuclear proteins, the Cyto-Fast™ Fix/Perm Buffer kit and True-Nuclear™ Transcription Factor Buffer kit (BioLegend) were used, respectively. Data acquisition was performed on a LSRII or LSRFortessa X-50 (BD Biosciences; Franklin Lakes, NJ, USA) cytometer. Data were analyzed using Kaluza v2.1 (Beckman Coulter; Indianapolis, IN, USA). Fluorochrome-labeled antibodies are listed in Supplementary Table S4. Flow cytometry gating strategies have been previously detailed [22].

### 2.9. Statistics

Data are reported as mean ± standard error of the mean (SEM). The statistical significance between two groups was determined via unpaired *t*-tests (Welch’s correction). The statistical significance of more than two groups was analyzed by one-way ANOVA with Dunnett’s multiple comparisons post hoc analysis. All statistical analyses were conducted by GraphPad Prism v10.1.2 (GraphPad Software; Boston, MA, USA). *p* values less than 0.05 were considered significant.

## 3. Results

### 3.1. Metabolomic Analysis of Human and Murine Plasma

**Figure 1:**
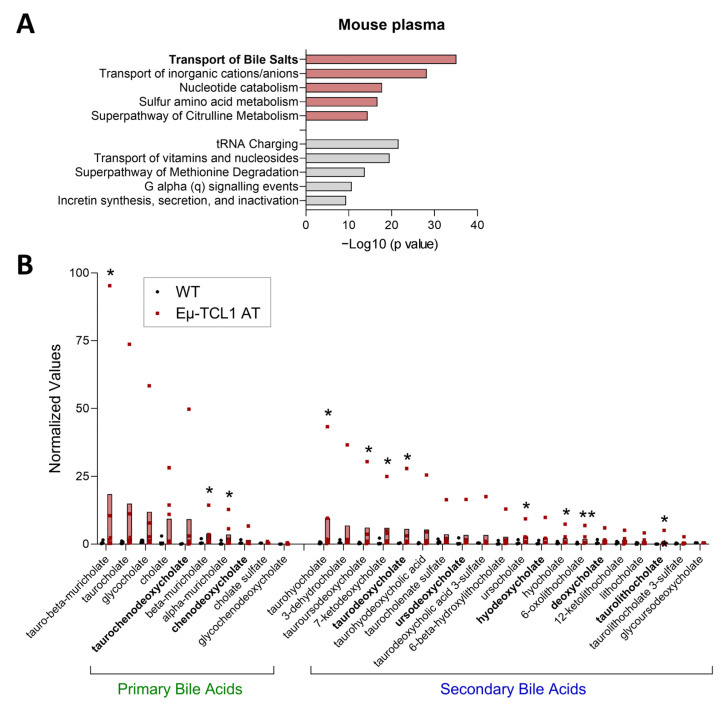
Untargeted metabolomics (UPLC–MS/MS; Metabolon Inc.) performed on plasma from Eµ-TCL1 adoptive transfer (AT) mice (n = 4) and C57BL/6 wild-type (WT) mice (n = 6). (**A**) Ingenuity Pathway Analysis of differential metabolites (Eµ-TCL1 AT vs. WT; 0.5 < log2FC < −0.5). Red = positive z-score; gray = negative z-score. (**B**) Abundance of bile acids in the plasma of WT and Eµ-TCL1 AT mice. Bolded text indicates key pathways or bile acids of interest. Asterisks denote significant difference from WT, as determined by Welch’s two-sample *t*-test. * *p* < 0.05, ** *p* < 0.01.

**Figure 2:**
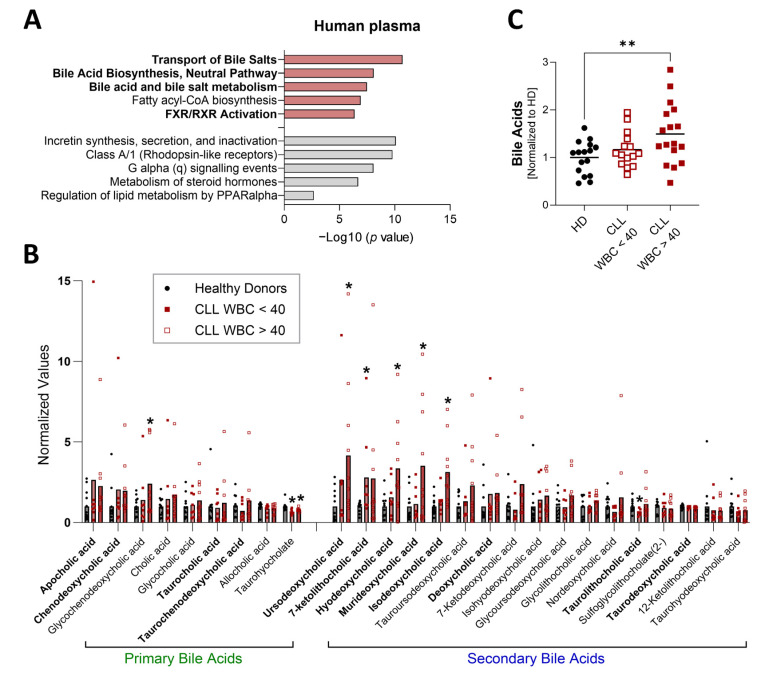
(**A**,**B**) Untargeted metabolomics (UPLC–MS/MS; NovoGene Co.) performed on plasma from CLL patients (n = 18) and healthy donors (HD; n = 12). (**A**) Ingenuity Pathway Analysis of differential metabolites (CLL vs. HD; 0.5 < log2FC < −0.5). Red = positive z-score; gray = negative z-score. Bolded text indicates bile acid-related pathways. (**B**) Abundance of bile acids (BAs) in the plasma of HD and CLL individuals. CLL patients are split into those with a white blood cell count (WBC) of less than the median of samples evaluated: 40,000 cells/µL blood (n = 8; WBC < 40) and those with a WBC greater than 40,000 cells/uL blood (n = 10; WBC > 40). Bolded BAs indicate those evaluated further in Figure 3 and Figure 4. (**C**) Fold difference in total BAs detected in plasma samples from CLL patients with WBC < 40 (n = 15) and CLL patients with WBC > 40 (n = 17), compared to healthy donors (HD; n = 16). Asterisks denote significant difference from HD, as determined by one-way ANOVA with Dunnett’s correction for multiple comparisons. * *p* < 0.05, ** *p* < 0.01.

To initially investigate metabolites that may contribute to TME immunosuppression in CLL, we employed the Eμ-TCL1 adoptive transfer (AT) murine model [23,24]. Untargeted metabolomics was performed on plasma from Eμ-TCL1 AT and age-matched wild-type (WT) mice (Figure 1, Extended Data File S1). Analysis of differential metabolites (Eμ-TCL1 AT vs. WT) identified bile acids (BAs) as a significantly altered metabolite family and “transport of bile salts” as one of the top pathways increased with CLL-like disease (Figure 1). Untargeted metabolomic analysis of human plasma samples similarly identified BAs as a significantly altered metabolite family and BA-related pathways as increased in CLL patients compared to age-matched healthy individuals (Figure 2A,B, Extended Data File S2). Total BA plasma levels were confirmed to be higher in CLL patients, especially those with greater tumor burden, compared to healthy controls (Figure 2C).

Similar primary and secondary BAs were elevated with CLL and CLL-like disease in human and murine plasma (Figure 1B and Figure 2B). Primary BAs were more significantly increased in the plasma of Eμ-TCL1 AT compared to WT mice, and secondary BAs were markedly heightened in CLL patients compared to healthy controls, potentially owing to differences in rate of disease expansion, liver enlargement, and intestinal microbiota [24,25,26]. Select differential BAs [per metabolomic analysis: apocholic acid (ACA), ursodeoxycholic acid (UCDA), 7-ketolithocholic acid (7KLCA), hyodeoxycholic acid (HDCA), murideoxycholic acid (MDCA), isodeoxycholic acid (IDCA)] and commonly abundant BAs (TCA, CDCA, TCDCA, DCA, TLCA, TDCA) were further evaluated for their impact on CLL cell proliferation and T-cell immunosuppression.

### 3.2. Cytotoxic and Immunodulatory Action of Bile Acids

**Figure 3:**
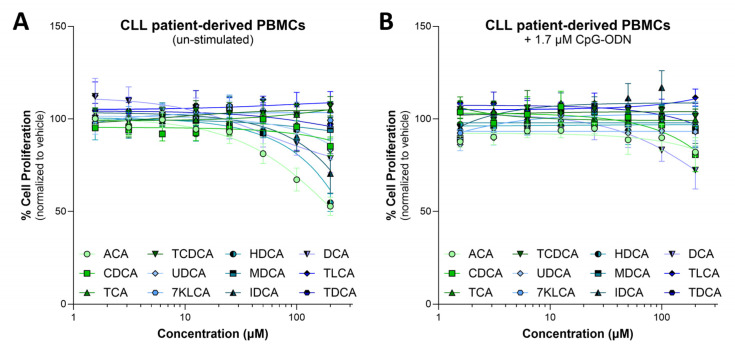
CLL patient-derived PBMCs (>90% CD19^+^/CD5^+^ cells; n = 4 patient samples) treated with 0–200 µM of bile acids for 48 h without (**A**) or with (**B**) the addition of 1.7 µM CpG-ODN 2006 (CpG-ODN). CLL cell proliferation was assessed by MTS assay. Results are demonstrated as percent proliferation, normalized to the vehicle control. ACA: apocholic acid, TCA: taurocholic acid, CDCA: chenodeoxycholic acid, TCDCA: taurochenodeoxycholic acid, UDCA: ursodeoxycholic acid, 7KLCA: 7-ketolithocholic acid, HDCA: hyodeoxycholic acid, MDCA: murideoxycholic acid, IDCA: isodeoxycholic acid, DCA: deoxycholic acid, TLCA: taurolithocholic acid, TDCA: taurodeoxycholic acid.

Most BAs were relatively non-toxic to CLL cells ex vivo (Figure 3). Interestingly, CLL cells were slightly more susceptible to BA-impaired growth when un-stimulated, compared to when they were stimulated with CpG-2006 oligonucleotides (CpG-ODN), a toll-like receptor 9 agonist commonly used to mimic TME immunostimulatory signals [27] (Figure 3).

**Figure 4:**
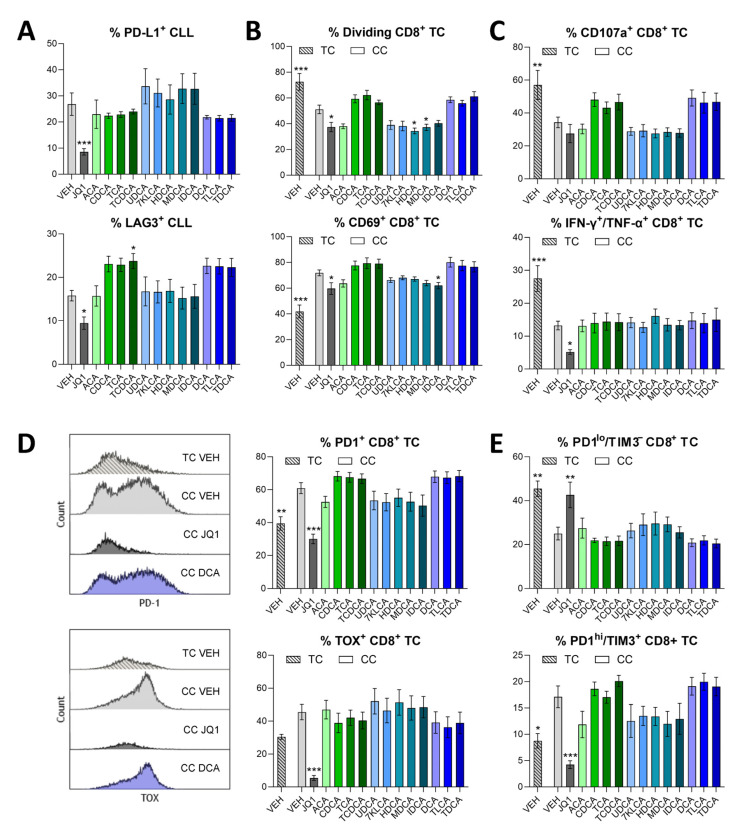
Healthy donor T-cells (TC) cultured alone or co-cultured (CC) with CLL patient-derived B-CLL cells (2:1 B-CLL:T) ratio, stimulated with 10 μg/mL plate-bound anti-CD3, 5 μg/mL soluble anti-CD28, and 1.7 μM CpG-ODN 2006, and treated with the indicated BAs (50 µM), control BET inhibitor (JQ1; 5 µM) or vehicle control (VEH; DMSO) for 48–96 h (n = 6 patient samples). Following treatment, cells were evaluated by flow cytometry for immune molecule expression and function. (**A**) Percentages of co-cultured CLL cells expressing immune inhibitory molecules (48 h culture). (**B**) Top: percentage of CFSE-stained CD8^+^ T-cells that underwent cell division (96 h culture). Bottom: percentage of activated (CD69^+^) CD8^+^ T-cells (48 h culture). (**C**) Following 48 h culture, cells were stimulated with PMA/ionomycin for 6 h with Brefeldin-A added for the final 5 h. Top: the percentage of CD8^+^ T-cells with membrane-localized CD107a. Bottom: the percentage of polyfunctional CD8^+^ T-cells co-expressing IFN-γ and TNF-α. (**D**,**E**) Percentages of CD8^+^ T-cells expressing immune inhibitory receptors or transcription factors (48 h culture). (**D**) Left panels illustrate representative flow cytometry plots for the expression of PD1 (**top**) and TOX (**bottom**) in CD8^+^ T-cells. (**E**) PD1^lo^/TIM3^−^ = progenitor-exhausted T-cells; PD1^hi^/TIM3^+^ = terminally exhausted T-cells. Data are represented as mean ± SEM. Asterisks denote significant difference from CLL or TC + B-CLL (CC) VEH (one-way ANOVA with Dunnett’s correction for multiple comparisons). * *p* < 0.05, ** *p* < 0.01, *** *p* < 0.001. ACA: apocholic acid, TCA: taurocholic acid, CDCA: chenodeoxycholic acid, TCDCA: taurochenodeoxycholic acid, UDCA: ursodeoxycholic acid, 7KLCA: 7-ketolithocholic acid, HDCA: hyodeoxycholic acid, MDCA: murideoxycholic acid, IDCA: isodeoxycholic acid, DCA: deoxycholic acid, TLCA: taurolithocholic acid, TDCA: taurodeoxycholic acid.

The BAs evaluated (at 50 µM—the lowest concentration shown to affect CD8^+^ T-cell function in previous studies [15]) demonstrated minimal effect on CLL-mediated immunosuppression (Figure 4, Supplementary Figure S2). Secondary BAs found to be differentially abundant in CLL patient plasma (UDCA, 7KLCA, HDCA, MDCA, IDCA) slightly increased PD-L1 expression on CLL cells, while common, but not differential, BAs (CDCA, TCA, TCDCA, DCA, TLCA, TDCA) slightly increased LAG3 expression on CLL cells (Figure 4A). Top differential primary and secondary BAs somewhat reduced the proliferation, activation (CD69 expression), and cytotoxic pore formation (CD107a membrane localization) of CLL-suppressed CD8^+^ T-cells, yet CD8^+^ T-cell inflammatory cytokine (IFN-γ and TNF-α) production was unaffected by any BA treatment (Figure 4B,C). The expression of inhibitory receptors, PD1 and TIM3, on CD8^+^ T-cells mirrored the effects of BA treatment on CLL PD-L1 expression (Figure 4A,D). However, neither significant shifts in the expression of the T-cell exhaustion-associated transcription factor, TOX, nor distribution of CLL-suppressed CD8^+^ T-cells into progenitor-like (PD1^lo^/TIM3^−^) and terminally (PD1^hi^/TIM3^+^) exhausted categories [28] were witnessed with any BA treatment (Figure 4D,E). Notably—while overall less impactful—5 µM of some BAs demonstrated a greater, yet non-significant, immunosuppressive effect (Supplementary Figure S3). At this lower dose, CDCA and DCA increased CD8^+^ T-cell TOX expression, and differential secondary BAs slightly increased the percentage of terminally exhausted CD8^+^ T-cells. Additionally, when cell cultures were treated with 150 µM BAs, the expression of both immune-activating and -suppressive factors somewhat decreased (Supplementary Figure S4).

## 4. Discussion

In this study, we found that BAs are differentially abundant in the peripheral blood of CLL patients and a murine model of CLL. These BAs do not appear to be cytotoxic to CLL cells or aid their proliferation at physiological concentrations. Immunomodulatory analyses did not solidify the impact of elevated BAs in CLL; however, BAs found to be differentially abundant in CLL patients displayed distinct immunomodulatory action (i.e., impaired CD8^+^ T-cell proliferation (Figure 4B) compared to less differential BAs. These findings suggest a potential influence of BAs on the immunosuppressive TME in CLL and warrant further investigation into the mechanistic influence of BAs on CLL pathogenesis. Previous studies have identified various metabolic alterations in CLL [29], including changes in acylcarnitine levels [30], sphingolipid metabolism [31], and the mevalonate pathway [32], but BA dysregulation has not been previously reported. Thus, our findings represent a novel contribution to the growing body of metabolomic research in CLL.

Several mechanisms may contribute to the observed elevation of BAs in CLL. Firstly, this phenomenon may be the result of an altered gut microbiome. Recent studies have demonstrated microbiome alterations in CLL [25,33]. Given the essential role of intestinal microbiota in converting primary BAs to secondary BAs, dysbiosis could contribute to the more pronounced elevation of secondary BAs observed in CLL patients. Additionally, CLL patients often exhibit hepatomegaly and liver infiltration [26], which could impact BA synthesis and metabolism. Furthermore, the recycling of BAs through the enterohepatic circulation may be disrupted in CLL due to alterations in BA transporters (e.g., NTCP, ASBT, OSTαβ) or signaling pathways that regulate this process. The dysregulation of BAs in CLL may also be interconnected with other reported metabolic alterations. Cholesterol metabolism produces the precursors for BA synthesis and is known to be altered in CLL [34]. Recent studies have highlighted the importance of cholesterol homeostasis and lipid raft dynamics in T-cell dysfunction in CLL [35], and BAs play a critical role in cholesterol homeostasis through their regulatory effects on cholesterol synthesis and catabolism [36]. This raises the intriguing possibility that BA elevation may be linked to broader lipid metabolic reprogramming in CLL, potentially contributing to both tumor cell survival and immune dysfunction [37].

Our ex vivo studies showed limited direct effects of BAs on CLL cell viability and T-cell function. However, within the complex TME in vivo, BAs may interact with other metabolites, immune cells, and stromal factors to influence disease progression. BAs act as signaling molecules that can be sensed by different receptors including FXR and GPBAR1 (TGR5), which are ubiquitously expressed and situated at the interface of the host immune system and the intestinal microbiota [32]. The BA-FXR axis has been shown to regulate TLR signaling and inflammasome assembly, acting as a brake for innate immunity [33,34]. FXR can also promote oncogenesis by modulating various factors like tumor suppressors (p53, p-Rb) and regulators of cell cycle (CDKs) and transcription (NF-kB, STAT3) [38,39,40]. Secondary BAs have been reported to be pro-tumorigenic in colorectal cancer and hepatocellular carcinoma by inducing stemness and senescence of tumor cells [41,42]. Beyond the gut, ectopic expression of FXR has been found to induce an immunosuppressive milieu characterized by exhaustive CD8^+^ T-cells and the heightened infiltration of myeloid-derived suppressor cells in lung cancer [43]. These findings suggest that the BA-FXR axis could potentially contribute to the immunosuppressive microenvironmental milieu in CLL as well.

Therapeutic interventions targeting BA metabolism or signaling may offer novel treatment approaches for CLL. Potential strategies could include pharmacological modulation of BA receptors, microbiome-targeted therapies, BA sequestrants, or combination approaches targeting BA signaling in combination with established CLL therapies to enhance disease management.

Several limitations of our study should be acknowledged. The relatively small number of CLL patients included in our analysis limits our ability to definitively associate BA levels with specific clinical features or outcomes. Future studies with larger patient cohorts encompassing different disease characteristics (e.g., indolent, aggressive, or relapsed CLL) are needed to validate our findings and explore potential correlations with clinical parameters. Our functional studies were conducted using isolated cells in culture, which may not fully recapitulate the complex interactions occurring in the CLL microenvironment in vivo, where the effects of BAs on CLL cell proliferation and T-cell function may differ. Although we discuss potential roles of BA receptors such as FXR and GPBAR1, we did not directly assess their expression or signaling in CLL cells or microenvironmental components.

Future studies are needed to address these limitations and further explore the mechanistic role of BAs in CLL pathogenesis. Specifically, future work should evaluate the presence of BAs in the supportive, tolerogenic niches to which CLL cells home (lymph nodes, spleen), and their impact on a broader sampling of TME bystander cells (e.g., nurse-like cells, myeloid cells) in addition to CLL cell survival signaling. To aid mechanistic understanding, the expression and function of BA receptors such as FXR and GPBAR1 in CLL cells and infiltrating immune cells, and the signaling pathways activated by BAs in these cells should be evaluated. Pharmacological modulation of BA metabolism or signaling in the Eμ-TCL1 AT murine model could further elucidate the impact of BAs on CLL progression. The potential interplay between BA metabolism and other metabolic alterations in CLL, such as changes in cholesterol homeostasis, sphingolipid metabolism, and mitochondrial function additionally warrants further investigation. Finally, assessing altered BA levels associated with response to common therapies or the advent of relapsed/refractory CLL could help determine the utility of BA-targeting agents in the treatment of CLL.

In conclusion, our identification of elevated BAs in CLL represents a novel finding that expands knowledge of metabolic alterations in this disease. While the precise role of BAs in CLL pathogenesis remains to be fully elucidated, our findings suggest that they may contribute to the immunosuppressive microenvironment of CLL and potentially influence disease progression. Further investigation of BA metabolism and signaling in CLL may yield new insights into disease biology and novel therapeutic approaches.

## Data Availability

Data will be made available from the authors upon reasonable request.

## Supplementary Materials

The following supporting information can be downloaded at: https://www.mdpi.com/article/10.3390/biomedicines13040874/s1, Supplementary Figure S1: Flow chart of study methods; Supplementary Table S1: Bile acid salts used; Supplementary Table S2: Characteristic of healthy donor samples used; Supplementary Table S3: Characteristics of CLL patient samples used; Supplementary Table S4. Flow cytometry antibodies and dyes used; Supplementary Figure S2: Additional information for immunomodulatory activity of bile acids at 50 µM; Supplementary Figure S3: Immunomodulatory activity of select bile acids at 5 µM; Supplementary Figure S4: Immunomodulatory activity of select bile acids at 150 µM; Extended Data File S1: Summary of murine plasma untargeted metabolomic data analysis; Extended Data File S2: Summary of human plasma untargeted metabolomic data analysis.
