# Supplementary material for: The Elevation and Impact of Peripheral Bile Acids in Chronic Lymphocytic Leukemia
Source: Biomedicines. 2025 Apr 4;13(4):874. doi: 10.3390/biomedicines13040874 (PMC12024544; doi:10.3390/biomedicines13040874)
Supplement: Supplementary file 1 [file biomedicines-13-00874-s001.zip › biomedicines-3526888 supplement.pdf]

Supplementary Methods and Data

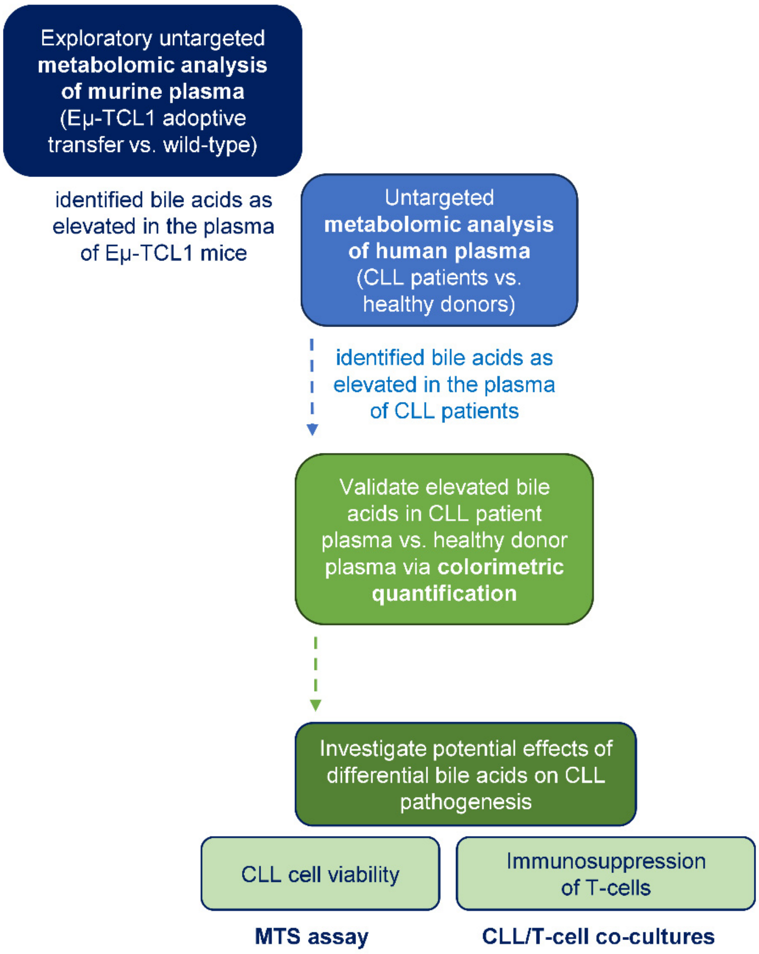

Supplementary Figure S1. **Flow chart of study methods**. Exploratory untargeted metabolomic analysis was first performed on murine plasma to determine differential metabolites in the blood of leukemic mice as compared to healthy mice. This experiment identified bile acids as elevated in the plasma of a murine model of CLL (Eμ-TCL1 adoptive transfer). We then performed similar metabolomic analysis of human plasma (CLL patients vs. healthy donors), which verified elevated bile acids was associated with CLL disease. This finding was validated by quantifying total bile acid levels in human plasma, using a colorimetric detection kit. These results prompted further investigation into potential effects of bile acids on CLL pathogenesis, such as CLL cell viability (determined by MTS assay) and immunosuppression of T-cells (evaluated using CLL/T-cell co-cultures).

Supplementary Table S1. Bile acid salts used.

Bile acid	Supplier	Catalog #
apocholic acid	Avanti Research	700241P
taurocholic acid	MedChemExpress	HY-B1788
chenodeoxycholic acid	MedChemExpress	HY-76847
taurochenodeoxycholic acid	MedChemExpress	HY-N2027
ursodeoxycholic acid	Fisher	501848203
7-ketolithocholic acid	MedChemExpress	HY-W018512
hyodeoxycholic acid	Sigma-Aldrich	H3878
murideoxycholic acid	MedChemExpress	HY-N0169B
isodeoxycholic acid	MedChemExpress	HY-W399297
deoxycholic acid	MedChemExpress	HY-N0593
taurolithocholic acid	MedChemExpress	HY-113308A
taurodeoxycholic acid	MedChemExpress	HY-B1899

Supplementary Table S2. Characteristic of healthy donor samples used.

Donor #	Sex	Age
1	Female	61
2	Female	62
3	Female	62
4	Female	70
5	Female	66
6	Male	60
7	Male	62
8	Male	64
9	Male	69
10	Male	61
11	Female	67
12	Male	66

Supplementary Table S3. Characteristics of CLL patient samples used.

CLL patient #	Sex	Age	Treatment status at sample date	IGHV[1] mutational status	FISH[2] Cytogenetics					Karyotype[3]
					Del 13q	Del 17p	Del 11q	Del 6q	Tri 12	Complex or Normal
1	Male	67	Naive	unknown	unknown	unknown	unknown	unknown	unknown	unknown
2	Female	73	Naive	unknown	unknown	unknown	unknown	unknown	unknown	unknown
3	Female	64	Naive	UNMUTATED	−	−	−	−	−	NORMAL
4	Female	66	Naive	UNMUTATED	+	−	+	−	−	unknown
5	Male	76	Naive	unknown	unknown	unknown	unknown	unknown	unknown	unknown
6	Male	69	Naive	UNMUTATED	−	−	−	−	−	NORMAL
7	Male	82	Naive	unknown	unknown	unknown	unknown	unknown	unknown	unknown
8	Male	71	Naive	UNMUTATED	−	+	−	−	−	unknown
9	Male	66	Naive	UNMUTATED	+	−	−	−	−	unknown
10	Male	68	Naive	UNMUTATED	−	−	−	+	−	unknown
11	Female	87	Naive	unknown	−	−	−	−	−	NORMAL
12	Female	70	Naive	UNMUTATED	+	−	+	−	−	unknown
13	Female	78	Naive	unknown	+	−	−	−	−	unknown
14	Male	64	Naive	unknown	unknown	unknown	unknown	unknown	unknown	unknown
15	Male	66	Naive	unknown	+	+	−	−	−	unknown
16	Female	70	Naive	UNMUTATED	−	−	−	−	+	unknown
17	Female	63	Naive	MUTATED	+	−	−	−	−	45,XX, add(11)(q25), del(13)(q12q22), -21[3]/ 46,XX[10]
18	Female	66	Naive	MUTATED	+	+	−	−	−	unknown

[1] IGHV: immunoglobulin heavy chain variable region
[2] FISH: fluorescence in situ hybridization
[3] Karyotype: Complex karyotype defined as harboring 3 or more chromosome abnormalities

Supplementary Table S4. Flow cytometry antibodies and dyes used.

Antigen	Clone	Fluorochrome	Supplier	Catalog #
CD101	BB27	PE/Cy7	BioLegend	331014
CD107a	H4A3	PE	BioLegend	328608
CD127	A019D5	Brilliant Violet 650	BioLegend	351326
CD19	HIB19	Brilliant Violet 605	BioLegend	302244
CD244	C1.7	Brilliant Violet 421	BioLegend	329532
CD44	C44Mab-5	Alexa Fluor 700	BioLegend	397522
CD69	FN50	Brilliant Violet 785	BioLegend	310932
CD8	SK1	Brilliant Violet 510	BioLegend	344732
GZMB	QA18A28	FITC	BioLegend	396404
IFN-γ	4S.B3	PE/Cy7	BioLegend	502528
IL-2	MQ1-17H12	APC	BioLegend	500310
LAG3	11C3C65	PerCP/Cy5.5	BioLegend	369312
PD-L1	29E.2A3	PE Dazzle	BioLegend	329732
PD-1	EH12.2H7	Brilliant Violet 421	BioLegend	329920
TCF1	7F11A10	PE	BioLegend	655208
TIM3	F38-2E2	Brilliant Violet 711	BioLegend	345024
TNF-α	MAb11	PE Dazzle	BioLegend	502946
TOX	TXRX10	eFluor-660	Thermo Fisher	50-6502-82
Zombie NIR™ Fixable Viability Kit			BioLegend	423106

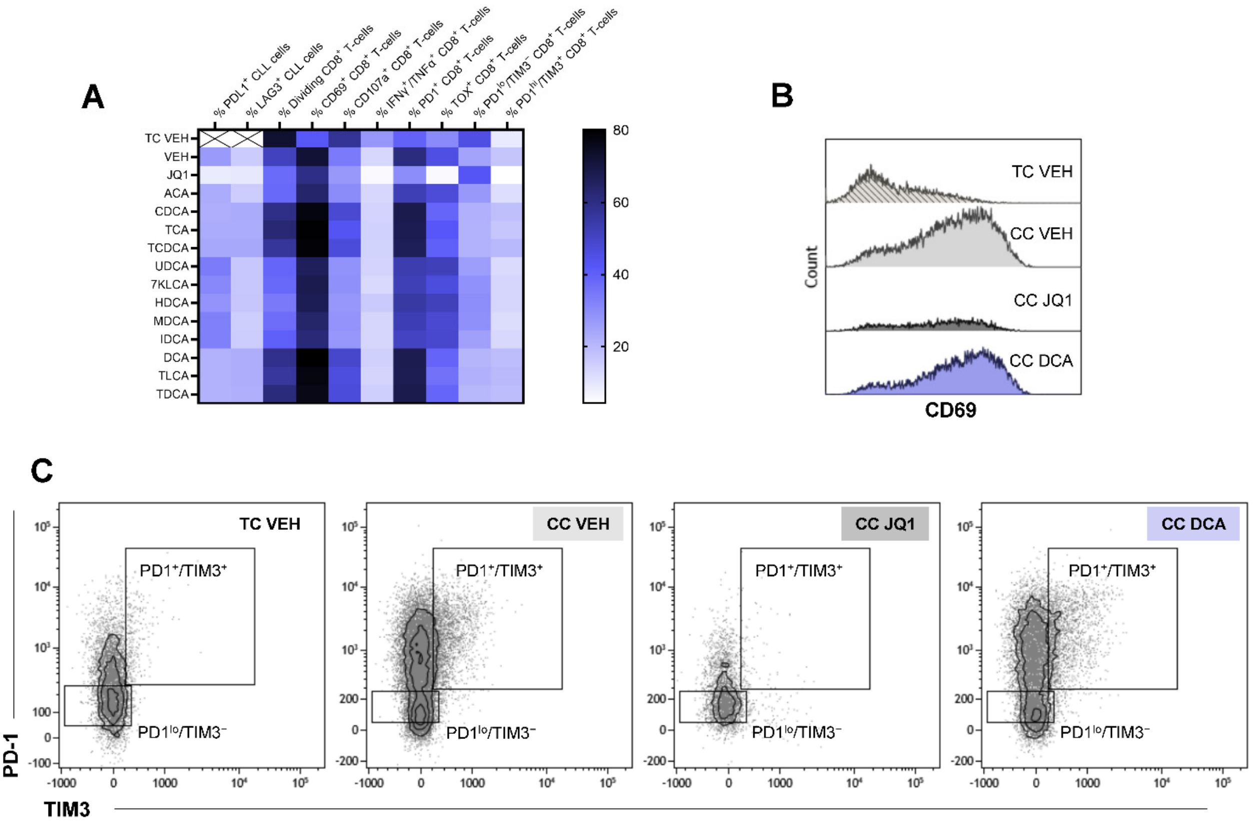

Supplementary Figure S2. **Additional information for immunomodulatory activity of bile acids at 50 µM (Figure 4).** Healthy donor T-cells (TC) cultured alone or co-cultured (CC) with CLL patient-derived B-CLL cells (2:1 B-CLL:T) ratio, stimulated with 10 µg/mL plate-bound anti-CD3, 5 µg/mL soluble anti-CD28, and 1.7 µM CpG-ODN 2006, and treated with the indicated BAs (50 µM), control BET inhibitor (JQ1; 5 µM) or vehicle control (VEH; DMSO) for 48-96 h (n=6 patient samples). Following treatment, cells were evaluated by flow cytometry for immune molecule expression and function. **(A)** Heatmap of average flow cytometry data. **(B)** Representative flow cytometry plots for the expression of CD69 on $CD8^+$ T-cells. **(C)** Representative flow cytometry plots for the identification of $PD\text{-}1^{lo}/TIM3^-$ (progenitor exhausted) and $PD\text{-}1^+/TIM3^+$ (terminally exhausted) $CD8^+$ T-cells. ACA: apocholic acid, TCA: taurocholic acid, CDCA: chenodeoxycholic acid, TCDCA: taurochenodeoxycholic acid, UDCA: ursodeoxycholic acid, 7KLCA: 7-ketolithocholic acid, HDCA: hyodeoxycholic acid, MDCA: murideoxycholic acid, IDCA: iso-deoxycholic acid, DCA: deoxycholic acid, TLCA: taurolithocholic acid, TDCA: taurodeoxycholic acid.

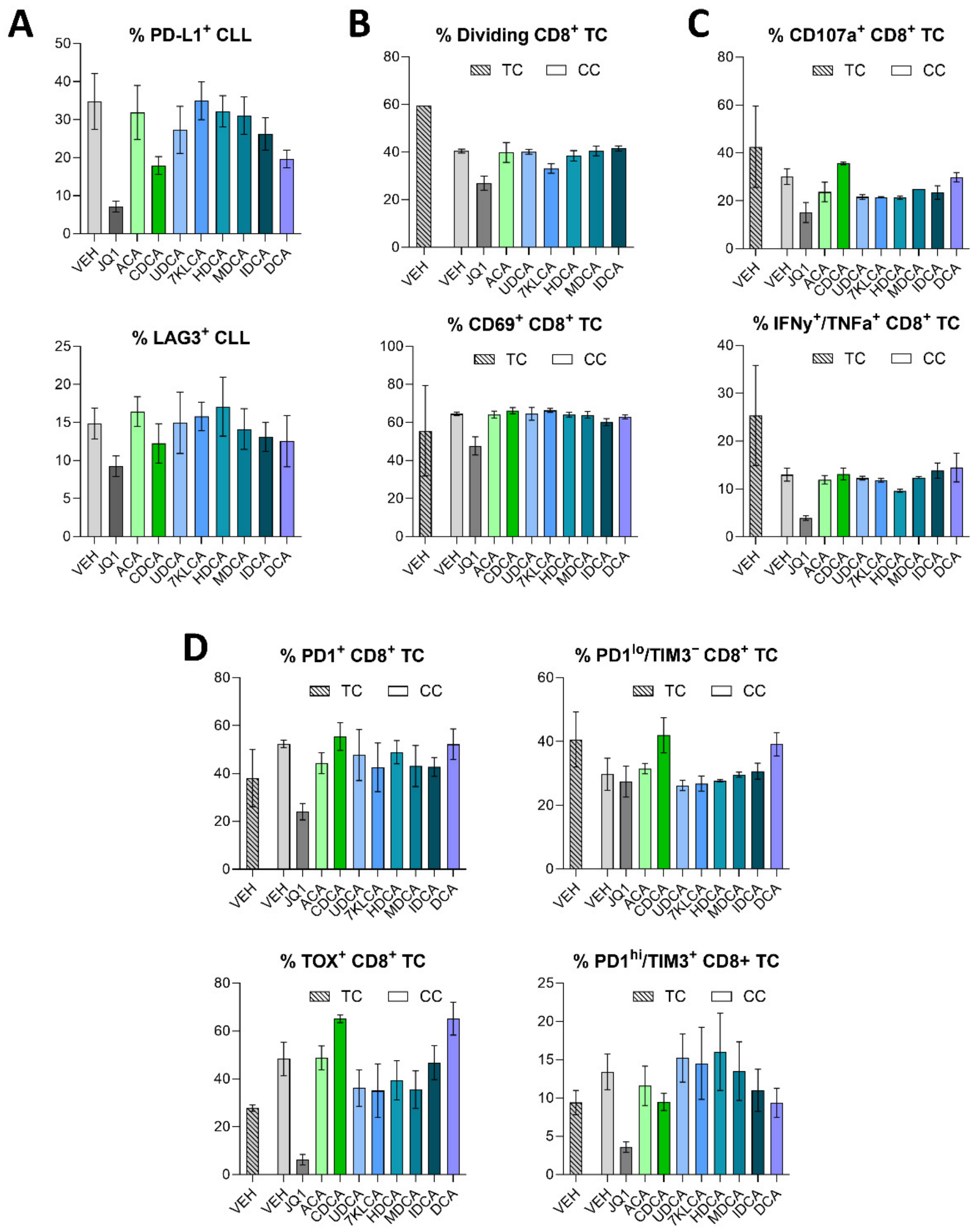

Supplementary Figure S3. Immunomodulatory activity of select bile acids at 5 μM. Healthy donor T-cells (TC) cultured alone or co-cultured (CC) with CLL patient-derived B-CLL cells (2:1 B-CLL:T) ratio, stimulated with 10 μg/mL plate-bound anti-CD3, 5 μg/mL soluble anti-CD28, and 1.7 μM CpG-ODN 2006, and treated with the indicated BAs (**5 μM**), control BET inhibitor (JQ1; 5 μM) or vehicle control (VEH; DMSO) for 48-96 h (n=4-6 patient samples). Following treatment, cells were evaluated by flow cytometry for immune molecule expression and function. **(A)** Percentages of co-cultured CLL cells expressing immune inhibitory molecules (48 h culture). **(B)** Top: percentage of CFSE-stained $CD8^+$ T-cells that underwent cell division (96 h culture). Bottom: percentage of activated ($CD69^+$) $CD8^+$ T-cells (48 h culture). **(C)** Following 48 h culture, cells were stimulated with PMA/ionomycin for 6 h with Brefeldin-A added for the final 5 h. Top: percentage of $CD8^+$ T-cells with membrane-localized CD107a. Bottom: percentage of polyfunctional $CD8^+$ T-cells co-expressing IFN-γ and TNF-α. **(D)** Percentages of $CD8^+$ T-cells expressing immune inhibitory receptors or transcription factors (48 h culture). PD-1^{lo}/$TIM3^-$ = progenitor exhausted T-cells; PD-1^{hi}/$TIM3^+$ = terminally exhausted T-cells. Data are represented as mean ± SEM. ACA: apocholic acid, CDCA: chenodeoxycholic acid, UDCA: ursodeoxycholic acid, 7KLCA: 7-ketolithocholic acid, HDCA: hyodeoxycholic acid, MDCA: murideoxycholic acid, IDCA: isodeoxycholic acid, DCA: deoxycholic acid.

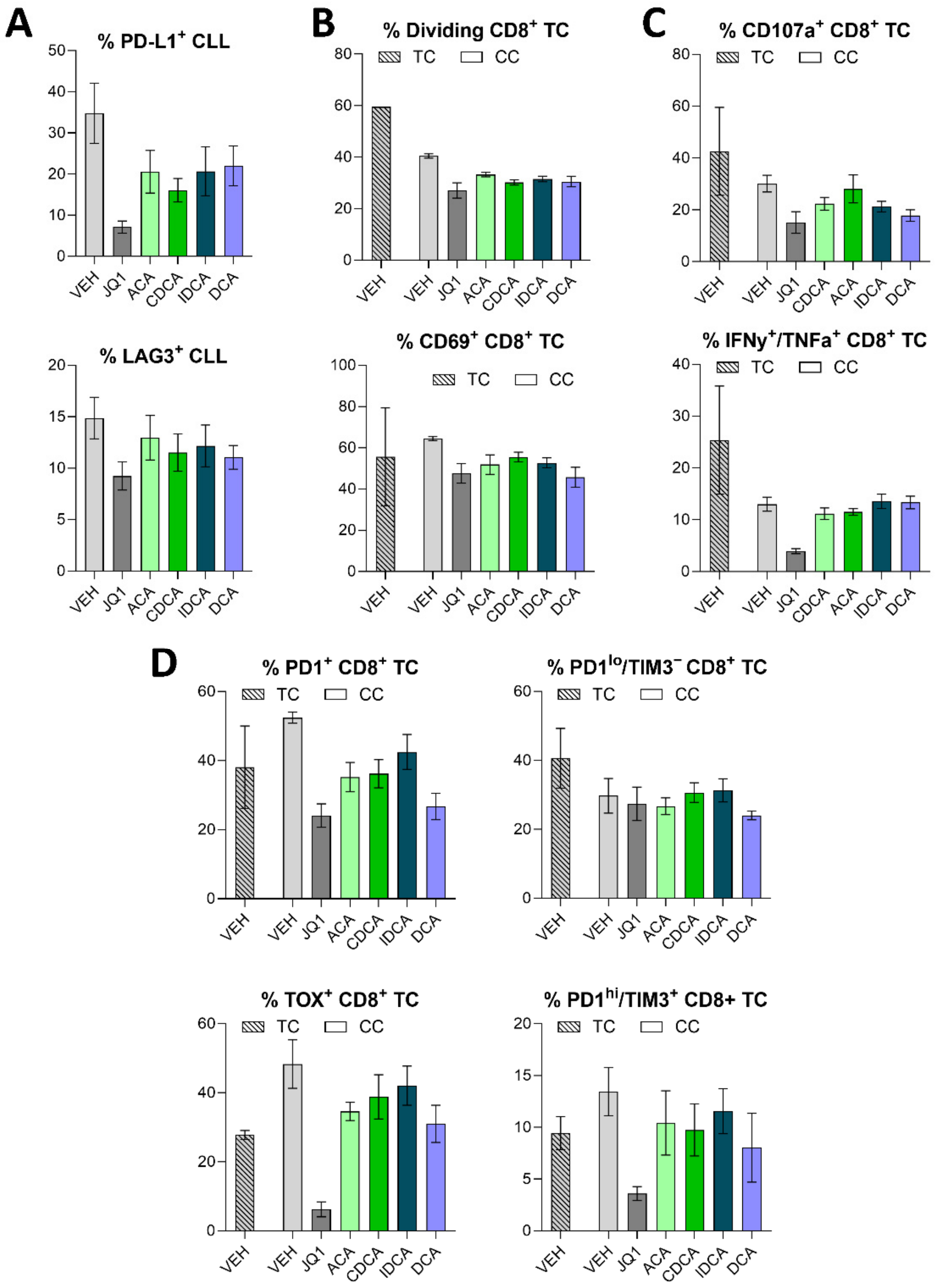

Supplementary Figure S4. Immunomodulatory activity of select bile acids at 150 μM. Healthy donor T-cells (TC) cultured alone or co-cultured (CC) with CLL patient-derived B-CLL cells (2:1 B-CLL:T) ratio, stimulated with 10 μg/mL plate-bound anti-CD3, 5 μg/mL soluble anti-CD28, and 1.7 μM CpG-ODN 2006, and treated with the indicated BAs (**150 μM**), control BET inhibitor (JQ1; 5 μM) or vehicle control (VEH; DMSO) for 48-96 h (n=4-6 patient samples). Following treatment, cells were evaluated by flow cytometry for immune molecule expression and function. **(A)** Percentages of co-cultured CLL cells expressing immune inhibitory molecules (48 h culture). **(B)** Top: percentage of CFSE-stained $CD8^+$ T-cells that underwent cell division (96 h culture). Bottom: percentage of activated ($CD69^+$) $CD8^+$ T-cells (48 h culture). **(C)** Following 48 h culture, cells were stimulated with PMA/ionomycin for 6 h with Brefeldin-A added for the final 5 h. Top: percentage of $CD8^+$ T-cells with membrane-localized CD107a. Bottom: percentage of polyfunctional $CD8^+$ T-cells co-expressing IFN-γ and TNF-α. **(D)** Percentages of $CD8^+$ T-cells expressing immune inhibitory receptors or transcription factors (48 h culture). PD-1^{lo}/$TIM3^-$ = progenitor exhausted T-cells; PD-1^{hi}/$TIM3^+$ = terminally exhausted T-cells. Data are represented as mean ± SEM. ACA: apocholic acid, CDCA: chenodeoxycholic acid, IDCA: isodeoxycholic acid, DCA: deoxycholic acid.
